# The Role of Community Centre-based Arts, Leisure and Social Activities in Promoting Adult Well-being and Healthy Lifestyles

**DOI:** 10.3390/ijerph10051948

**Published:** 2013-05-10

**Authors:** Mat Jones, Richard Kimberlee, Toity Deave, Simon Evans

**Affiliations:** 1 Department of Health and Applied Social Studies, University of the West of England, Bristol, BS16 1DD, UK; E-Mail: richard.kimberlee@uwe.ac.uk; 2 Centre for Child & Adolescent Health, University of the West of England, Bristol, BS8 2BN, UK; E-Mail: toity.deave@uwe.ac.uk; 3 Institute of Health & Society, University of Worcester, Worcester, WR2 6AJ, UK; E-Mail: simon.evans@worc.ac.uk

**Keywords:** community development, arts and health, healthy living, holistic programmes, well-being

## Abstract

Developed countries are experiencing high levels of mental and physical illness associated with long term health conditions, unhealthy lifestyles and an ageing population. Given the limited capacity of the formal health care sector to address these public health issues, attention is turning to the role of agencies active in civil society. This paper sought to evaluate the associations between participation in community centre activities, the psycho-social wellbeing and health related behaviours. This was based on an evaluation of the South West Well-being programme involving ten organisations delivering leisure, exercise, cooking, befriending, arts and crafts activities. The evaluation consisted of a before-and-after study with 687 adults. The results showed positive changes in self-reported general health, mental health, personal and social well-being. Positive changes were associated with diet and physical activity. Some activities were different in their outcomes—especially in cases where group activities were combined with one-to-one support. The results suggest that community centre activities of this nature offer benefits that are generically supportive of health behaviour changes. Such initiatives can perform an important role in supporting the health improvement objectives of formal health care services. For commissioners and partner agencies, accessibility and participation are attractive features that are particularly pertinent to the current public health context.

## 1. Introduction

Developed countries are experiencing high levels of mental and physical illness associated with long term health conditions, unhealthy lifestyles and an ageing population. These are interconnected issues: for example cardiovascular disease, diabetes, obesity, and drug and alcohol use are linked with common mental health problems such as anxiety and depression and, in some cases, severe mental health conditions [1]. Furthermore these issues disproportionately affect lower socio-economic groups, and significantly contribute to the widening gap in health inequalities [2].

Formal health care services have both limited capacity and ability to address the challenges of an ageing population and multiple chronic morbidities [3]. In this context attention is increasingly turning outside the formal health care sector to the role of wider civil society and, in particular, community and voluntary sector agencies in the promotion of health and well-being [4,5,6,7]. Agencies that work in community centre settings may be well placed to respond to this agenda. While their specific characteristics vary, in the context of this paper, community centres are multi-purpose organisations dedicated to the provision of services in their neighbourhood or highly local catchment area [8]. Run by community management committees, voluntary agencies or, on occasions, local government authorities, they deliver a wide range of group and individually focused activities. In recent years in the UK, many community centre-based organisations have explicitly linked their aims to the promotion of health and wellbeing through the delivery of, for example exercise, cooking, social, arts and creative activities [9]. Some primary health care providers have reciprocated with the “social prescribing” of these activities to their patients [10]. Advocates [5,11] suggest that community based organisations are well placed to offer informal and user-led activities that fit with local interests and provide additional benefits, such as a basis for developing social support networks and civic action.

However there has been limited research on the impact of such community engagement activities on health related behaviours [12,13]. A positive association between participation in community based activities and health is documented at national (e.g., [14]) and local levels (e.g., [15,16]). There is evidence of the health effects of highly structured and specified interventions in community settings that are delivered as an adjunct to healthcare services. Limits to resources often mean that these interventions need to be targeted at high risk groups and have eligibility criteria that restrict access. At a larger policy scale considerable research has been undertaken to examine the impact of area-based neighbourhood renewal and civil society programmes [17,18]. Such initiatives have built upon evidence of the links between social capital and health and are concerned with population-wide impacts—or with the impact on population segments such as socio-economically disadvantaged groups. Much of the research on health care services and area-based programmes include community-centre activities as a partial or peripheral concern. In contrast, this paper seeks to position community centre initiatives as a central focus of enquiry through the examination of a mix of community based group activities for adults [12,19].

Drawing upon a programme in the South West of England UK, the main objective of this paper is to investigate the associations between participation in community centre-based activities and the health and wellbeing of adults. It then seeks to analyse associations between specific aspects of the programme and identified outcomes. The final objective is to consider the implications of the results for the development of community centre health and wellbeing initiatives.

## 2. South West Well-Being Programme

The South West Well-being (SWWB) programme ran from 2008–2011 and was developed and delivered by a consortium of fifteen community-based voluntary sector organisations from across the South West of England, UK. It was funded by the Big Lottery, local government and local healthcare commissioning bodies, all of whom had a role in appraising the rationale, design and quality of the programme. It also received in-kind support through volunteers. The stated aim of the programme was to “support the healthy living and well-being of individuals and communities by providing locally accessible, people-focused and holistic approaches to tackle health inequalities particularly for those people most in need” [20]. Central characteristics of the programme were:

1. A focus on individuals’ experiencing low level mental ill health, long term health conditions, low levels of physical activity and/or diet related ill-health. These criteria were combined with low income and/or social isolation.
2. The provision of a holistic service that focuses on the social aspects of health and well-being: being inclusive, fun, non-judgemental and non-threatening; people-centred and self-directed lifestyle change-depending on their needs and wishes.
3. A focus on reducing stress and anxiety, increasing physical activity and healthy eating, through confidence building and encouraging the development of friends, social networks and local community participation.
4. A focus on local collaboration with other agencies and stakeholders with an interest in health promotion and building community capacity.

One consortium organization held overall governance and commissioning accountability for the programme. Lead practitioners and agency managers met every three months to monitor and review programme delivery. In order to adopt common standards of delivery and share best practice, practitioners from all project sites attended three training sessions and translated programme-wide guidance into detailed curricula and schedules for each activity. All groups were led by practitioners with basic community development and health promotion training and were often supported by volunteers. Some group sessions were supplemented with a structured course in individual counselling or personal health goal setting. The types of activities delivered under the programme were classified into three themes—mental wellbeing, physical activity and healthy eating—according to their primary focus. The promotion of social wellbeing was often an underlying, if not central, objective for many activities. Mental wellbeing groups focused on art and craft activities and friendship-building activities. Examples included a “group textile project” to create a knitted representation of a community picnic; and a “moving-on group” that offered practitioner and peer-led advice for people who had a history of poor mental health and social isolation. Physical activity groups included a focus on gentle exercise, dance and aerobic training. Examples included a “chair exercise group” for people with mobility difficulties; and an introductory “belly dancing class”. Health eating groups focused on dietary health. Examples included a “grow and cook group” where an instructor taught recipes based upon produce from a community garden; and a “weight management group” where participants with a history of over-weight problems offered peer support alongside dietary advice from a health promotion practitioner.

Typically the group activities were scheduled as ten week blocks of two hour sessions. Within a shared framework, implementation of the programme was highly tailored to the circumstances of each community centre. Some agencies delivered the programme through multi-purpose community centres that offered health, social care, child care, housing, education, debt advice and other services. Other agencies worked through the premises of partner agencies and community groups. While the specific contexts were diverse, the activities shared a focus on higher social deprivation neighbourhoods or areas—such as rural settings—that lacked other community-based services.

## 3. Methods

### 3.1. Procedure

The present study reports on the research with adults taking part in ten SWWB of the fifteen projects. These were the first ten projects to commence programme delivery in the early part of 2008. Eligible study participants were over 18 years old, newly registering with the community centre and taking part in at least one SWWB funded activity. We employed a study recruitment “window” of approximately three months with the objective of recruiting between 50 to 80 study participants by list registration entry at each of the ten project sites.

The baseline administration of the questionnaire took place as participants began substantive engagement with the project. The follow up administration of the questionnaire took place either at the end of their engagement or at the completion of an activity period, for example the end of a twelve week course of activities. The average interval between baseline and follow-up completion was 110 days (range: 41–190; SD: 41).

The questionnaire was either administered by a member of the research team or, following standardised training and advice, by project workers directly. To address the potential for a social desirability bias, participants self completed the questionnaire after having first been advised that their responses would be confidential to the researchers, processed anonymously and would have no bearing on their opportunities to take part in project activities. On occasions, the administrator would assist where there was reading or literacy difficulties.

### 3.2. Ethical issues

Participants were provided with written and verbal information on the study and written consent was requested. Administrators made it clear that participants had the right to not participate or to withdraw at any point. The study was given ethical approval by the Faculty of Health and Life Sciences Research Ethics Committee of the University of the West of England, Bristol.

### 3.3. Questionnaire Tool (SWWBQ)

Designed to be used for before-and-after evaluation, the South West Well-being Questionnaire (SWWBQ) consisted of sets of validated and original measures that covered general health, social well-being, personal well-being, mental ill health, healthy eating, and physical activity. The inclusion of mental ill health, healthy eating and physical activity constructs followed the SWWB programme’s emphasis on these aspects of behavioural change. The core elements of SWWBQ were adopted from a national Well-being questionnaire. This was developed in 2007–2008 specifically for all initiatives funded under the Big Lottery’s Well-being Programme [21]. The main measures are summarised in Table 1. Five point Likert scales were used for most items in the tool.

**Table 1:** Summary of the measures covered in the SWWBQ.

General health: *How would you describe your health generally over the last week?* (5 point scale, widely used measure)	
Social Well-Being scale (6 items, 5 point scales, SWB-6; adapted from European Social Survey Round 3)	
*Below are some statements about feelings and thoughts. What best describes your experience of each over the past four weeks?(paraphrased)*	
**	*I feel like I belong to something I call community*
**	*There are people in my life who really care about me*
**	*People in my local area help one another*
**	*I regularly meet socially with friends and relatives*
**	*I find it easy to meet people who share my hobbies or interests*
	*I often help with or attend activities organised in my local area*
Center for Epidemiological Studies Depression scale (7 items, 5 point scales, CES-D-7 [22])	
*Below are a number of things people might say that they feel. How often during the past week would each description have applied to you? (paraphrased)*	
**	*You felt happy or contented*
**	*You felt depressed*
**	*You felt engaged or focused in what you were doing*
**	*You felt energised or lively*
**	*You felt lonely*
**	*You felt everything you did was an effort*
	*Your sleep was restless*
Warwick Edinburgh Mental Well-being Scale (7 items, 5 point scales, WEMWBS-7 [23])	
*Below are some statements about feelings and thoughts. What best describes your experience of each over the past four weeks?(paraphrased)*	
**	*I’ve been feeling optimistic about the future*
**	*I’ve been feeling useful*
**	*I’ve been feeling relaxed*
**	*I’ve been dealing with problems well*
**	*I’ve been thinking clearly*
**	*I’ve been feeling close to other people*
	*I’ve been able to make up my own mind about things*
Mental well-being: life satisfaction. *All things considered, how satisfied are you with your life as a whole nowadays?* (10 point scale, widely used measure)	
Healthy eating (5 point scales)	
	Fruit & vegetable intake: portions per day (widely used measure)
	Enjoyment: *I enjoy eating healthy food* (study specific question)
	Importance: *I value putting effort and care into the food I eat* (study specific question)
Physical activity	
	GP Physical Activity Questionnaire (5 point scales, GPPAQ [24])
	Enjoyment: *I enjoy taking part in regular physical activity* (5 point scale, study specific question)
	Importance: *Regular physical activity is important for my health* (5 point scale, study specific question)

The social well-being scale was based on a set of established 6 items assessed for validity and reliability in the European Social Survey Round 3 and Big Lottery’s Well-being Programme [21].

1. Belonging (community) I feel like I belong to something I call community
2. Support (intimate) There are people in my life who really care about me
3. Support (community) People in my local area help one another
4. Engagement (social) I regularly meet socially with friends and relatives
5. Engagement (intimate) I find it easy to meet people who share my hobbies or interests
6. Participation (community) I often help with or attend activities organised in my local area

An initial version of the tool was piloted with one SWWB project in 2008 with adults aged between 25 and 60. Participants reported that the questions were acceptable and straightforward to understand. Post analysis of the study data indicated acceptable internal consistency for the scales:

1. CES-D-7 baseline Cronbach’s α 0.853; follow-up Cronbach’s α 0.715
2. WEMWBS-7 baseline Cronbach’s α 0.885; follow up Cronbach’s α 0.849
3. SWB-6 baseline Cronbach’s α of 0.714; follow-up Cronbach’s α of 0.708

In addition, the questionnaire included demographic questions concerning age band, gender, race/ethnicity, disability, domestic circumstances, care responsibilities, employment states and entry route onto the activity.

### 3.4. Data Analysis

The questionnaire data were entered onto a specially designed Microsoft Access database. The data were then exported and analysed using SPSS 19. Data checks against hard copy questionnaires were run to ensure the reliability of the data entry. Where at least half the items of an instrument scale were present the technique of person mean substitution (where the imputed value for a variable with missing data is derived from the non-missing items for that person) was adopted to impute data [25]. For the overall analysis, group outcomes were compared using a parametric (paired *t*-test) statistical test. Pearson chi-squared was used for further tests of association in the dataset.

## 4. Results

### 4.1. Recruitment of Study Participants

**Figure 1:**
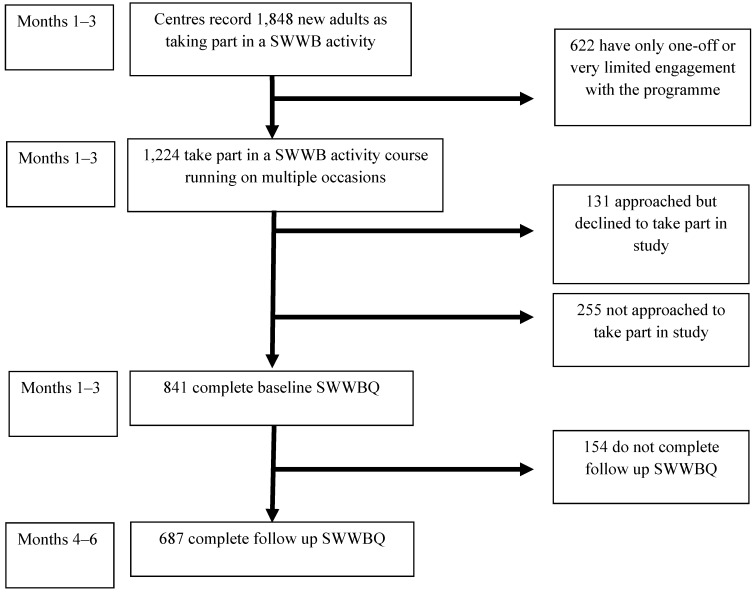
Flow diagram for study participants.

During the study period, 687 adults completed both baseline and follow-up questionnaires. Figure 1 shows how this group relates to the wider population of adults accessing SWWB programme activities. This shows that of those taking part in a SWWB activity course, 56% completed both study questionnaires (687/1,224). Of note at the baseline data collection stage, 255 individuals were not approached to take part in the study. These were individuals enrolling at popular project sites where the study recruitment target was met at an early point in the data collection period. Individuals who were approached but declined to complete the questionnaire (131/1,224) included those who could not commit the time, did not wish to report personal information, or had recently completed other similar forms. Figure 1 also shows how, out of 1,848 adults recorded as having contact with the programme centres, about one third (622) had only very limited engagement. This group was not approached to participate in the study, given that their exposure to the programme was likely to be considerably more limited than those that had enrolled with specific group activities.

### 4.2. Participant Profile

The summary in Table 2 shows that the participants were well represented in all age groups, 91% (n = 625) were White, 78% (n = 536) were female, 33% (n = 228) lived alone and just 25% (n = 171) were in employment, training or education. The number of respondents from each of the ten community centres ranged from 48 to 93 (mean = 62.5). Although much of the activity participation combined a range of elements as part of a holistic approach to promoting wellbeing, 33% participants (n = 230) took part in an initial project activity that had a focus on healthy eating (Table 2). Thirty-four percent (n = 235) of participants engaged in group activities that had additional structured individual practitioner support sessions and the rest (n = 452) engaged in group activities that only had informal, unstructured individual practitioner support.

**Table 2:** Socio-demographic and project activity profile of participants (N = 687).

Variable		N	(%)
**Gender**			
	Male	179	(26)
	Gender	536	(78)
**Age band**			
	18–30	102	(14.8)
	31–40	138	(20.2)
	41–50	159	(23.1)
	51–60	91	(13.2)
	61–70	128	(18.6)
	70+	61	(8.9)
	Not known	8	(1.2)
**Race/ethnicity**			
	White (British & Other)	623	(90.7)
	Black or Minority Ethnic Group	38	(5.5)
	Not known	26	(3.8)
**Employment status**			
	Training, education	42	(6.1)
	Retired	209	(30.4)
	Seeking work	136	(19.8)
	Full time family carer	88	(12.8)
	In employment	129	(18.8)
	Incapacity for work (illness, disability)	62	(9)
	Not known	21	(3.1)
**Domestic circumstances**			
	Living alone	228	(33.2)
	Living with partner & children	132	(19.2)
	Living with partner only	136	(19.7)
	Living with other relative	54	(7.9)
	Living children only	44	(6.4)
	Not known/Other	93	(13.6)
**Main focus of initial project activity**			
	Physical activity	144	(21.0)
	Mental wellbeing	101	(14.7)
	Healthy eating	230	(33.5)
	Befriending	147	(21.4)
	Non-specific	65	(9.4)
**Format of initial project activity**			
	Group activities plus a structured series of 1-1 practitioner support sessions	235	(34.2)
	Group activities plus unspecified or *ad hoc* 1-1 practitioner contact	452	(65.8)
**Main route for activity enrolment**			
	Project direct contact (e.g., leaflet, practitioner contact)	201	(29.2)
	Informal networks (e.g., word of mouth)	197	(28.7)
	Healthcare agency (e.g., GP, primary care centre)	176	(25.6)
	Other agency (e.g., social services, children’s centre)	85	(12.4)
	Not known	28	(4.1)
**Additional registration notes**			
	Practitioner referral notes recorded depression, anxiety or another mental health issue	111	(16.1)
	Practitioner referral notes recorded obesity or overweight, BMI > 25	178	(25.9)
	Participants recorded being in receipt of a disability benefit	106	(15.5)
	Participants recorded being unpaid carer for an adult with illness or disability	54	(7.9)

Project monitoring records suggest that the 687 participants had a similar socio-demographic profile, in terms of gender, age-band, race/ethnicity, to the non-study population (n = 1,161/1,848) that used the community-centre services during the study period.

### 4.3. Health and Well-Being Results

Results of the paired *t*-tests for the baseline and follow-up measures are described in Table 3. The results demonstrate that there were statistically significant changes for all of the overall measures (<0.05) for improved general health; social well-being; mental well-being; enjoyment and importance of heating eating; general physical activity; and enjoyment and importance of physical activity. There was a significant reduction in self-reported mental ill health. These changes were associated with participation at baseline and follow-up stages in the SWWB programme, and reflect the central areas of focus for the initiative.

An exception in this positive overall shift was self-reported fruit and vegetable consumption where there was a reduction of 0.36 portions (SEM 0.19, *t* 3.08, df 585, *p* < 0.002) at follow-up. This was a whole group level change. Section 4.4.2 reports on further analysis to investigate an association between fruit and vegetable consumption and the healthy eating specific focus of some activities.

The social wellbeing scale is reported as a global score as well as with its individual items (Table 3). Although the overall positive changes were highly significant (<0.001), no statistically significant changes were found for the item (v), *people who share my hobbies and interests* and item (mean difference 0.016, SD 1.39, SEM 0.05, *t* 0.28, df 640, *p* = 0.77) and (vi), *people in my life who really care about me* (mean difference 0.01, SD 1.01, SEM 0.04, *t* −0.19, df 654, *p* = 0.85). These represent more intimate aspects of social engagement and support, compared to the other items covered by the social well-being scale.

**Table 3:** Health and wellbeing results: Paired *t*-tests. N = 687.

Measure	Baseline Mean (SD ^a^)	Follow up Mean (SD)	Mean Difference (SD)	Standard Error of the Mean (SEM)	*t* value (df ^c^)	*p* value
General health	2.60 ^b^ (0.95)	3.12 ^b^ (1.02)	0.51 (1.07)	0.41	12.34 (675)	<0.001 ^h^
Social wellbeing: SWB-6 ^d^	20.05 (3.16)	21.82 (4.18)	1.77 (4.07)	0.17	−10.52 (633)	<0.001 ^h^
*i. Relatedness (community: belonging)*	3.00 (1.16)	3.32 (1.12)	0.32 (1.16)	0.05	−6.90 (645)	<0.001^ h^
*ii. Engagement (community: attending local activities)*	2.99 (1.63)	3.36 (1.59)	0.37 (1.55)	0.06	−6.07 (648)	<0.001^ h^
*iii. Engagement (community: meeting friends & family)*	3.71 (1.08)	3.91 (0.99)	0.20 (1.11)	0.04	−4.59 (648)	<0.001^ h^
*iv. Support (community: local people help)*	3.33 (0.98)	3.51 (0.99)	0.02 (1.39)	0.72	−4.45 (648)	<0.001^ h^
*v. Engagement (intimate: sharing interests & hobbies)*	2.70 (1.13)	2.72 (1.19)	0.02 (1.39)	0.05	0.28 (640)	0.77^ j^
*vi. Support (intimate: people care about me)*	4.32 (0.85)	4.33 (0.89)	0.01 (1.01)	0.04	−0.19 (654)	0.85^ j^
Mental well-being: life satisfaction	6.17 (2.40)	7.01 (2.17)	0.84 (2.67)	0.10	−8.00 (661)	<0.001^ h^
Mental well-being: WEMWBS-7 ^e^	23.24 (5.24)	25.52 (4.48)	2.28 (5.08)	0.21	−10.48 (621)	<0.001^ h^
Mental ill health: depression CES-D-7 ^f^	10.93 (4.83)	8.94 (2.96)	−1.99 (4.58)	0.20	−8.29 (601)	<0.001^ i^
Healthy eating: fruit & vegetable intake	3.90 (2.18)	3.54 (2.12)	−0.36 (2.85)	0.19	3.08 (585)	0.002^ i^
Healthy eating: enjoyment	3.75 (0.97)	4.19 (1.01)	0.44 (1.00)	0.03	−9.87 (662)	<0.001^ h^
Healthy eating: importance	3.76 (0.96)	4.20 (0.99)	0.44 (1.20)	0.05	−9.233 (668)	<0.001^ h^
Physical activity: GPPAQ ^g^	1.93 (1.05)	2.90 (1.12)	0.97 (1.36)	0.05	−18.17 (651)	<0.001^ h^
Physical activity: enjoyment	3.16 (1.71)	3.73 (1.15)	0.56 (1.64)	0.03	−8.02 (639)	<0.001^ h^
Physical activity: importance	3.52 (1.2)	3.79 (1.18)	0.27 (1.46)	0.02	−2.92 (353)	0.004^ h^

*Notes*: ^a^ SD: Standard Deviation. ^b^ Units of value for baseline and follow up scores are derived from 5 point Likert scales where 1 = lowest and 5 = highest. ^c^ df: degrees of freedom. ^d^ SWB-6: Social Well-being 6 item scale. ^e^ WEMWBS-7: Warwick Edinburgh Mental Well-being Scale, 7 item scale. ^f^ CES-D-7: Center for Epidemiological Studies Depression, 7 item scale. ^g^ GPPAQ: General Practitioner Physical Activity Questionnaire. ^h^ Significant increase the measure. ^i^ Significant reduction in the measure. ^j^ No significant change in the measure.

### 4.4. Further Analyses

#### 4.4.1. Associations between Well-Being, Healthy Eating and Physical Activity

Aspects of mental well-being were strongly linked to attitudes towards healthy eating and physical activity. At follow up people reporting higher mental well-being (WEMWBS-7 score) were more likely to:

1. Enjoy eating healthy food (χ^2^ 52.794, *p* < 0.001)
2. Value putting effort and care into food (χ^2^ 56.507, *p* < 0.001)
3. Enjoy taking part in regular physical activity (χ^2^ 31.640, *p* = 0.007)
4. Believe that physical activity is important for health (χ^2^ 46.185, *p* < 0.001)

#### 4.4.2. Associations between Outcomes and the Focus of Activity Input

There were associations between the focus of project inputs and the behaviour changes. Thus activities that focused on healthy eating were more strongly associated with positive changes in self reported fruit and vegetable consumption, in contrast to other project activities with no specific healthy eating focus (χ^2^ 33.649, *p* < 0.001). Activities focused on physical exercise were associated changes in GPPAQ scores compared to those that did not have physical activity as their primary focus (χ^2^ 79.227, *p* = 0.03). Mental health focused projects were associated with a greater change in mental wellbeing scores compared to those did not have this specific focus (χ^2^ 36.305, *p* < 0.001). There was a similar association between befriending focused activities and social well-being (χ^2^ 17.744, *p* < 0.001).

#### 4.4.3. Outcomes Associated with Format of Activity: One-to-One and Group Support

Participant outcomes were analysed by the format of the activity. Structured individual practitioner support was more strongly associated with positive mental health (χ^2^ 19.358, *p* < 0.001) and physical activity (χ^2^ 37.368, *p* = 0.02) compared to those who only obtained informal and unstructured individual practitioner support. This difference was still evident after controlling for age and gender.

#### 4.4.4. Outcomes Associated with Self *vs.* Practitioner Referral

There were a number of routes through which participants came to enrol on programme activities. A majority were, in effect, self referred: they enrolled via word of mouth or direct project contacts. Others were referred or recommended by a healthcare agency (25.6%) or other agency (12.4%). The latter participants were more likely to be out of employment, carers or be entitled to disability benefits. Those referred or recommended started with lower health and well-being scores and were more strongly associated with improvement across mental health (χ^2^ 11.700, *p* < 0.02) and social wellbeing (χ^2^ 8.493, *p* = 0.037) measures compared to those who were self referred.

## 5. Discussion

A range of adult age groups participated in the South West Well-being (SWWB) project activities although there was a predominance of females and people out of employment. There was a positive set of associations between participation in community centre-based activities and the aspects of health and well-being measures that respondents reported on. The separate measures for health and psycho-social wellbeing were closely associated with one another, and there was some evidence that the impacts were associated with the focus and format of the inputs and the referral routes.

The results of the study suggest that community centre activities offer benefits that are supportive of health and well-being. A number of features of the activities may help to account for these positive changes. Centres delivering the project activities had close referral and recommendation relationships with healthcare and other partner agencies, although informal routes also played an important role in accessing the services. As Rankin *et al.* [11] found, the programme activities were developed to connect with locally defined interests and sought to embed activities in participants’ everyday lives. Framing activities in terms of fun, leisure, creativity and socialising—as opposed to illness prevention and the amelioration poor health—is likely to have contributed to the appeal and accessibility of the activities. There are close associations between health-related lifestyle behaviours, such as poor diet and low physical activity, which lends support to a holistic service model. It indicates that work in one area can deliver wider benefits for participants. This case is reinforced for participants where unhealthy lifestyles are compounded with low income, social isolation and other forms of social exclusion [26]. Although the results support, overall, the idea that health-related lifestyle behaviors are closely connected, some findings point to complexity in this regard. Notably the self reported reduction in fruit and vegetable intake countered the pattern of results. Sub-analysis showed that this was not the case for individuals who participated in activities with a specific focus on healthy eating. For the sample as a whole, the result may reflect baseline social approval bias of the measure. This can either be seen as a drawback in the programme design or that it reduces the strength of association between dietary and other health-related behaviours.

There are a range of implications of this study for agencies, practitioners, commissioners and researchers. Commissioners and partner agencies—particularly in the formal healthcare sector—can look to the wider health promoting role of community centres [6,27]. Generic health promoting services in a community context may act as a supplement or alternative to mainstream healthcare provision, especially where alternative approaches are absent. The multiple routes for enrolment on to community centre-based activities are likely to address unmet health needs, while the development of new and potentially supportive social networks provide local assets for health.

The study clearly demonstrates that National Health Service (NHS) and other agencies were able to connect individuals with health and social needs to project activities as part of a targeted referral or recommendation process. However, it was also evident that the services attracted adults with no professionally assessed health needs. Further research is needed to understand the benefits, drawbacks and cost-effectiveness of programmes that adopt this type of open-access approach given an argument that services may fail to reach target groups with high health needs. This raises the question of the extent to which such community centre activities are likely to narrow or widen inequalities in health outcomes and health care access. It is important to note that this study reports only the experiences of adults who enroll on to SWWB activity groups. During the study period, this was about one third of service users. Further research is needed to understand the characteristics and perspectives of individuals who have more limited contact with community centres, not least because it remains hard to interpret the significance of such contact.

The high profile of well-being in local policy making means that it will be important for members of commissioning groups to have a broad understanding of what contributes to, and impacts on, improved well-being and to extend consideration beyond more narrow conceptions of health. Activities such as those delivered through the community centres in this study have a part to play in helping shift an agenda towards the promotion of wellbeing and positive health.

The study has a number of limitations which have a bearing on the interpretation of the findings. Due to limited research funds, the study design did not employ a comparison group, which would have offered a clearer basis for determining the intervention effects. The study was only able to test associations between factors—although the further analyses examined the relationships between project inputs and their anticipated impacts. The follow-up period only captured change on completion of participation in the project activities; we were not able to undertake further data collection to explore longer term behavioural changes. Although the study focused on new entrants, a longer period of data collection might also have informed an understanding of the effects of ongoing and sporadic participation. We explored differences in format, focus and referral patterns, however the project activities were heterogeneous which meant that it was hard to identify further characteristics of the interventions that might account for the results. This is an issue arising from the research context: diverse, emergent and informal structures are defining and inherent characteristics of many community centre activities.

## 6. Conclusions

This study found that group-based activities in community centres are associated with improvements in the health and wellbeing of adults who experience poor health and other forms of social disadvantage. Although other relevant work exists on the behavioural effects of primary healthcare interventions and comprehensive area-based initiatives in community settings, previous research has not made community centre schemes the focus for enquiry. The South West Well-being programme illustrates an initiative that sought to deliver a community focus on promoting capacity for personal and social well-being, as opposed to the management of ill health and disease. This reflects a reorientation towards promoting healthy behaviours and well-being, and pro-active investment to avoid future ill health costs.

From a wider public health perspective, the study indicates a need for a more strategic approach to the delivery in community centre services. Although local gains can be made in the short- to medium-term, it is likely that greater benefits can come from comprehensive and coordinated commissioning of community-based wellbeing services [9]. There is also scope for greater transfer of knowledge and innovation in service delivery between community projects that have much share from their locally developed expertise.
